# High-Efficiency, Wide Working Bandwidth Antenna Based on SOI Platform for Optical Phased Array

**DOI:** 10.3390/mi12080996

**Published:** 2021-08-21

**Authors:** Zihao Wang, Jiali Liao, Yixiang Xie, Yanling Sun, Xifeng Li, Wei Li

**Affiliations:** 1 School of Physics and Optoelectronic Engineering, XiDian University, Xi’an 710071, China; wangzihao@stu.xidian.edu.cn (Z.W.); lixifeng@stu.xidian.edu.cn (X.L.); wli0805@stu.xidian.edu.cn (W.L.); 2 State Key Laboratory of Pulsed Power Laser Technology, Hefei, Anhui 230037, China; 3 China Helicopter Research and Development Institute, Jingdezhen 333001, China; xyx2864490@126.com

**Keywords:** nano-optics, integrated optics, optical antenna, optical phased array

## Abstract

A novel structure of a subwavelength surface optical antenna for optical phased array is proposed in this paper. An asymmetric vertical grating structure is applied to achieve high emission efficiency (73% at 1550 nm). Optical antennas with large fabrication tolerances can also maintain a wide working bandwidth of 1 dB between 1350 and 1850 nm. The far-field scanning characteristics of 16-channel optical phased array are investigated in this study by employing the proposed antenna. The results show that the background suppression without considering side lobes caused by the antenna arrangement is −24.5 dB when the phase difference is 0 and when the scan range is as large as ±14.8° × 73.6°.

## 1. Introduction

OPAs have attracted great attention with the application of light detection and ranging (LIDAR) in remote sensing imagery and autonomous vehicles. Optical phased arrays (OPAs) based on a silicon platform are considered the primary solution for LIDAR because of their low manufacturing cost, high integration, and compatibility with CMOS technology. As application requirements have increased, OPAs have evolved toward small-sized, low power consumption and large-scale on-chip electrical/thermal–optical subsystem arrays. However, the ensuing difficulties are high power consumption and control complexity. A considerable amount of excellent work has been conducted by researchers for these concerns [1,2,3]. One approach is to combine beam wavelength modulation in one dimension with phase modulation in the other dimension, thus reducing the number of phase shifters from N^2^ to N to achieve reduced power consumption and simplified control. However, according to the literature [4,5,6,7,8,9,10], the antenna of this kind is limited by the fact that the working bandwidth is not wide enough to achieve beam modulation in short wavelength ranges only.

In most silicon on insulator (SOI) OPAs, grating couplers are used as optical antennas, and their low output power limits the performance of OPAs due to the low transmission efficiency of grating couplers [9,10,11,12,13]. Many efforts have been made to improve the transmitting efficiency of OPA antennas [14,15,16,17,18]. This includes some excellent optical antenna arrays that achieved good performance with low loss and a high side-lobe level (SLL). The optical antenna in the SOI OPA LIDAR is a grating coupler fabricated on a waveguide (periodically etched waveguide), a method that slightly avoids diffraction of light toward the substrate, resulting in an optical antenna whose emission efficiency barely exceeds 55%, according to a seminal published report [13]. High optical antenna emission efficiency of more than 90% has been obtained by increasing the antenna size to 3 mm while significantly increasing the antenna pitch size. However, the structural complexity of the antenna OPA system of this design, especially the highly complex electrical/thermal–optical part, leads to small fabrication tolerances and a narrow working wavelength bandwidth [16]. In addition, researchers also proposed the combination of dual-etch and apodization design approaches that a grating based on such a structure can achieve a very high fiber coupling efficiency of 85% [19].

In general, large OPA systems usually use thermal/electrical–optical hybrid modulation and wavelength modulation, which requires a wide 3 dB bandwidth or even a 1 dB bandwidth under certain conditions. How to optimize the optical antenna in order to improve the transmitting efficiency, scanning performance, and working bandwidth, thereby improving the far-field of OPA, is an important issue. To date, the existence of unidirectional guided resonances (UGRs)—resonances radiating to only one side of the photonic crystal plate, with no mirror layer placed on the other side—has been demonstrated [20]. This has inspired the study of asymmetric vertically etched grating couplers. Single angle tilt-etched waveguide grating couplers have also been studied, and many outstanding works have been verified and demonstrated that tilt-etched waveguide grating couplers can provide high efficiency [21,22,23].

In this paper, a novel optical antenna with a double-angle, tilt-etched grooved asymmetric vertical structure and subwavelength surface grating on SOI substrate is proposed, which can provide a wide working bandwidth. By optimizing the structural parameters of the antenna, the diffraction to the substrate is effectively suppressed, and the emission efficiency is improved. In addition, we discuss the fabrication tolerance of the proposed optical antenna in detail, and according to the analysis of the calculation results, the OPA composing this antenna has better far-field scanning characteristics.

## 2. Structure

**Figure 1:**
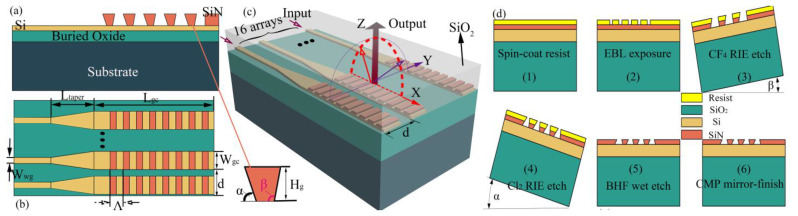
Schematic diagram of the structure and processing process of the proposed antenna. (**a**) Side view of the antenna; (**b**) top view of the antenna; (**c**) schematic of the antenna for optical phased arrays of integrated light detection and ranging (LIDAR) on silicon on insulator (SOI) substrate; (**d**) the essential step-by-step flow chart of the fabrication process. EBL, electron-beam lithography; PECVD, plasma-enhanced chemical vapor deposition; CMP, chemical–mechanical polishing [20].

Figure 1a shows a side view of the optical antenna on the SOI substrate, from bottom to top from the vertical direction. The dark green area is the Si substrate; the light green area is the 2 or 3 μM buried SiO_2_ layer; and the yellow and orange areas are the 0.22 μM Si and SiN layers, respectively, both covered with SiO_2_ layers, which are not drawn for easy observation. This design also makes the proposed optical antenna surface grating, which can increase the length of the grating, improve the emission efficiency of the optical antenna, reduce the full width at half maximum (FWHM) of the Gaussian-like light emitted by the optical antenna, and thus improve the spatial resolution [24].

The optical antenna element consists of three parts, as shown in Figure 1b. The first part is the input waveguide, which has a width of 0.5 μM. A silicon waveguide with this width can transmit transverse electric (TE) fundamental mode light in the wavelength range of 1350–1850 nm [25]. The guided mode passes through it into the tapered mode converter before its phase or wavelength is adjusted by a phase modulator or a tunable laser. The second part is the tapered mode converter, which helps to reduce the transmission loss caused by the difference in the effective refractive index between the first and third parts. The transmitting region (grating coupler) is the third part, which is used to couple out the optical energy, where the SiN low refractive index layer is made into a surface grating structure, unlike the conventional SOI optical grating antenna.

Our aim was to achieve a larger scale aperture antenna, as this would reduce the beam width of the far-field main flap, thereby providing higher spatial resolution. However, due to the high refractive index contrast of typical silicon waveguides formed by Si (*n* = 3.48) and SiO_2_ (*n* = 1.44), large-scale gratings require very shallow etched Si surfaces (5–15 nm). Therefore, the emitting region is obtained from strip-line surface gratings, and a CMOS-compatible alternative is the introduction of the low-index dielectric material SiN_x_ (*n* = 1.875) to form the gratings [24,26]. According to the phase matching condition, the grating diffraction is governed

(1) $$k_{0}n_{eff}=k_{0}n_{c}\sin\theta +q\frac{2\pi }{\Lambda }$$

where *k_0_* = 2 π/*λ*, *n_c_* is the refractive index of the air (equal to 1), *n_eff_* is the effective index for the optical mode in the grating, *θ* is the diffraction angle, *q* is an integer representing the diffraction order (equal to 1 in our discussion), and Λ is the period of the waveguide grating. Let *λ* = *λ*_i_, *θ* = *θ_i_*; Equation (1) becomes:

(2) $$\sin\theta_{i}=n_{eff}-\frac{\lambda_{i}}{\Lambda }$$

where *λ_i_* is the input waveguide mode working wavelength. From Equation (2), the $n_{eff}-\frac{\lambda_{i}}{\Lambda }$ calculation result of the equation is between −1 and 1, which can be considered as the wavelength that can be emitted. The bandwidth of the grating coupler will increase as the *n_eff_* decreases, a conclusion that has been demonstrated in previous studies [27]. Therefore, in addition to the increase in antenna size, we can expect that the bandwidth will increase if the surface grating coupler efficiency can be greatly increased.

A vertically asymmetric grating structure is used in the antenna to achieve higher coupling efficiency and reduce transmit power losses. Tilt etching has not yet been used for optical antennas but has been well studied in fabrication processes [20,21,22,23,28,29,30,31,32]. We refer in detail to the fabrication steps provided in [20] and give a schematic step-by-step process flow diagram of our proposed antenna structure in Figure 1d. To improve the emission efficiency of the optical antenna, the SiN and Si layers are placed very close to each other, which means that the etching time needs to be precisely controlled during the fabrication process. Although the method poses significant processing challenges, it is worthwhile if the performance of optical antennas based on this asymmetric structure can be improved.

## 3. Simulation and Discussion

Simulations were performed to study the pattern profile, field propagation, emission efficiency, and working bandwidth by using a three-dimensional finite-difference time-domain (3D FDTD) method [33]. The emission efficiency is defined as the ratio of the energy emitted into the antenna free space to the energy entering the antenna. With the help of the FDTD solution, the structural parameters of this optical antenna are optimized, and its performance in terms of working bandwidth and emission efficiency is demonstrated in this section. In addition, the scanning characteristics of the array this antenna is composed of are investigated in this section.

### 3.1. Antenna Element

**Figure 2:**
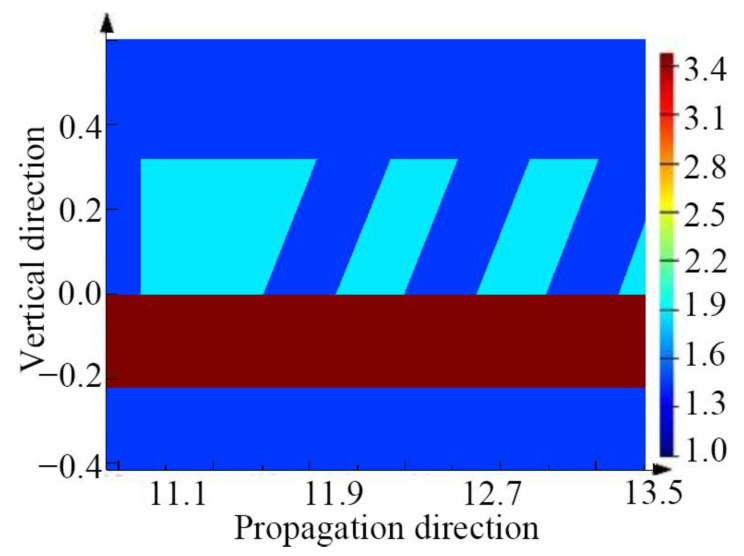
The partial refractive index profile of the antenna.

To ensure a clear representation of the optical antenna, the refractive index distribution of the antenna cross-section is shown in Figure 2, which shows part of the grating structure.

A TE polarized beam is set as input, and two power monitors are employed to measure the proportion of emission and leakage part of the total input power. The parameters to be optimized and their specific meanings are shown in Table 1, and these parameters are additionally given in Figure 1b. In addition, in Table 1, we also list the symbols and the meanings of these parameters.

**Figure 3:**
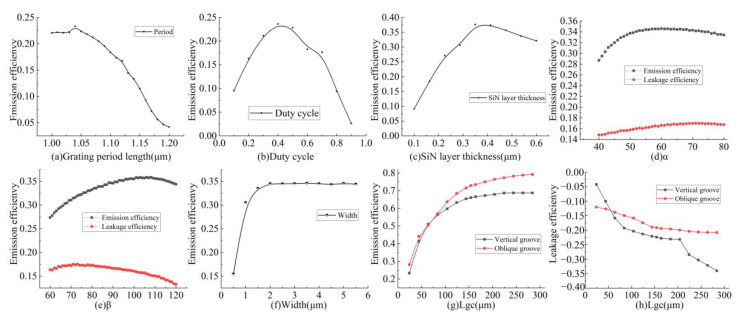
Optimization results of parameters affecting the emission efficiency of the proposed antenna. (**a**) The emission efficiency in relation to grating coupler period length; (**b**) the emission efficiency in relation to grating coupler duty cycle; (**c**) the emission efficiency in relation to SiN layer distance with Si waveguide; (**d**) the emission efficiency in relation to grating coupler etch α angle; (**e**) the emission efficiency in relation to grating coupler etch β angle; (**f**) the emission efficiency in relation to grating area width Wgc; (**g**) curve of grating area length Lgc versus emission efficiency and its comparison with the emission efficiency of vertically etched antenna; (**h**) curve of grating area length Lgc versus leakage efficiency and its comparison with the emission efficiency of vertically etched antenna.

The period Λ of the grating is simulated and optimized according to Equation (1), and the results are shown in Figure 3a. The emission of the antenna is highest at 1550 nm when Λ is 1.04 μM. The duty cycle of the etched period is optimized to 0.4 at Λ of 1.04 μM, as shown in Figure 3b. We further investigated the effect of the thickness of the SiN layer on the emission efficiency, as shown in Figure 3c, and the results show that the highest emission efficiency is achieved at a SiN thickness of 0.35 μM. In particular, the two angles of the grating slot are optimized as shown in Figure 3d,e, respectively. The emission efficiency of the antenna is highest at 1550 nm when *α* is 55° and *β* is 110°. After completing the optimization of the above structural parameters, the length and width of the antenna were investigated.

As can be seen in Figure 3f, the curve of the emission efficiency saturates after the waveguide width increases to 2 µM, and there is almost no growth in the emission efficiency during the change in width from 2 to 5.5 µM. Taking into consideration the width of the antenna and the transmitting efficiency, the antenna width of 2 µM was chosen. The effect of the length of the antenna on the transmitting efficiency was then studied, comparing the red curve representing the tilted etching method with the black curve representing the vertical etching in Figure 3g,h. It can be observed that the designed antenna has a slightly lower transmitting efficiency than that of the conventional antenna at a shorter transmission distance, but as its length grows, its transmitting efficiency grows with a slight decrease, but it keeps growing continuously compared with when the conventional antenna transmitting efficiency has leveled off. This means that the downward leakage of the conventional transmitting antenna is larger, and the designed transmitting antenna effectively reduces the leakage loss.

Subsequently, we investigated the effect of the taper length of the grating region and input waveguide used to connect the antenna on the transmission efficiency of the antenna and set its length to 11 µM.

**Figure 4:**
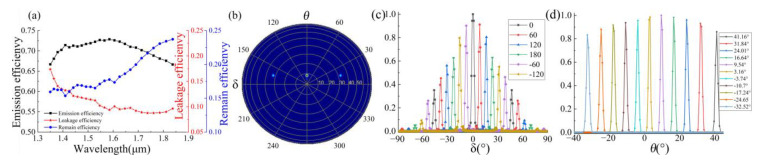
Scanning characteristics of OPA composed of the designed antenna based on 1 dB working wavelength range. (**a**) The 1 dB bandwidth of the antenna; (**b**) Far-field radiation simulation result of 16-optical antenna array; (**c**) Far-field radiation simulation result by considering each channel keeps ideal phase difference controlled by phase shifters; (**d**) Normalized optical output profile in the far-field as the beam was swept in the *θ* axis by changing the working wavelength from 1350 to 1850 nm.

Based on the optimized structure parameters listed in Table 1, we calculated the emission bandwidth of the optical antenna, as shown in Figure 4a. It can be observed that the 1 dB working bandwidth of the designed antenna is 500 nm from 1350 to 1850 nm. The leakage efficiency is also shown in Figure 4a, and it can be noted that the proportion of energy leaked at the central wavelength is significantly reduced, which also shows that this waveguide structure can effectively suppress the downward light energy leakage and improve the emission efficiency. In addition, the remaining efficiency is also shown in Figure 4a, which indicates that there is energy remaining in the emission area with *Lgc* at 150 µM, and the transmitting efficiency can be improved by increasing the antenna length (this is consistent with the results in Figure 3g).

The far-field characteristics of an optical antenna element or array are obtained by monitoring the near-field to far-field transformation [34]. Monitoring the power monitor in the near-field gives the near-field emission, and by using the near-field to far-field transformation, the swept FOV can be obtained. Due to the need to simulate the far-field effect for the antenna of 16 arrays, the length of the antenna coupling area *Lgc* is reduced to 40 µM in this part of the simulation. However, this result affects the half-height width of the beam of the antenna emitting far-field and the emission efficiency. The effect of light field scanning can be clearly demonstrated.

For a wavelength of 1550 nm, the diffraction angle *θ* of a single optical antenna is 9.53°, as shown in Figure 4b. The FWHM is directly related to the length of the antenna. A longer antenna corresponds to more grooves. As the *Lgc* of the antenna increases, the FWHM can be effectively reduced, as the transmitting efficiency of the antenna increases. By using the proposed surface grating antenna, OPA with higher spatial resolution can be obtained.

Owing to the same wavelength waveguide mode, adjacent elements keep the same phase difference by controlling the phase of the input source (which equals to being controlled by phase shifters), coupling to each antenna. The scanning angle *δ* is given by

(3) $$\sin\delta =\frac{\lambda \Delta \varphi }{2\pi d}$$

where *d* is the distance between adjacent elements and is set to be 1.6 μM by considering all factors. It can be observed that the antenna array can sweep ±14.8° when the additional phase Δ*φ* changes from −180° to 180°.

We further investigates the longitudinal sweeping characteristics of the array. In this case, steering of the emission angles is achieved by adjusting the working wavelengths, with the same phase of each element then set to 0. The emission angle *θ* is given by Equation (2), which can help to achieve a wide sweeping angel range of 73.6° from −32.5° to 41.1° in the longitudinal direction, as shown in Figure 4d. In the case of wavelength modulation without considering the side-lobe brought by the antenna arrangement, according to the simulation results, the antenna can achieve a background rejection rate of −24.5 dB.

### 3.2. Fabrication Tolerance Analysis and Comparison with Other Work

**Figure 5:**
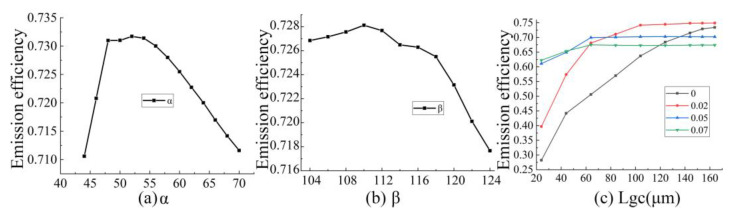
The fabrication tolerance in relation to grating coupler etches at *α* and *β* angle. (**a**) The emission efficiency of the proposed antenna under the condition that *α* has 5–10% error with the exact value; (**b**) the emission efficiency of the proposed antenna under the condition that *β* has 5–10% error with the exact value; (**c**) transmitting efficiency of the proposed antenna for different over-etching depths.

In contrast to previous work [21,22,23,28,29,30,31,32], the proposed antenna design incorporates double-angled grooves, which makes it difficult to manufacture. In this section, we analyze the fabrication tolerance of the oblique angle groove in detail. The most critical point is to study the effect on the emission efficiency in the case of errors in the two tilt angles. Figure 5a shows the transmitting efficiency for an error of ±10° in *α*, and we observed that if the remaining parameters are accurate, the emission efficiency of the antenna is in fact very little affected by *α*. When there is an error of ±10° in the design value of *β*, the reduction in emission efficiency is minimal. This means that the proposed antenna has a very good tolerance for the fabrication of double-angled grooves.

In addition, the proposed antenna structure may also be affected by over-etching, for which an investigation was carried out. The results are shown in Figure 5c, which illustrates that the transmitting efficiency of the antenna increases when the over-etching value is small (less than 0.02 µM, error of less than 5% of the total etch depth of 0.35 µM). When the over-etching value is too large, the transmitting efficiency of the antenna decreases; it can be considered that the increase in the over-etching value will lead to the antenna transmitting efficiency to reach the saturation value corresponding to the smaller antenna length. Based on the design concept of surface grating, the etching accuracy needs to be improved to avoid the surface antenna being affected by over-etching.

By comparing the data listed in Table 2, it can be seen that the antenna proposed in this paper is able to significantly increase the working bandwidth of the antenna. Although the energy emission efficiency is slightly less, as the length of the antenna increases, it is able to have higher emission efficiency according to Figure 3g.

## 4. Conclusions

In this work, we proposed a new type of surface optical antenna for OPA and optimized its emission efficiency through simulation. The optical antenna achieved a high emission efficiency of up to 73% at 1550 nm due to its up–down asymmetric structure and a wide working wavelength 1 dB bandwidth from 1350 to 1850 nm.

In addition, we discussed the far-field overview of a 16-channel OPA by employing an optical antenna. It was able to achieve a scanning range of ±14.8° × 73.6°, and the antenna allowed the OPA to achieve better scanning far-field characteristics. This approach shows promise as a means of achieving high emission efficiency for other applications, such as imaging sensors, OPA LIDAR, and free-space communications.

## Figures and Tables

**Table 1:** Parameters optimization results of the optical antenna.

Name	Meaning	Result
L_gc_ (μM)	Grating area length	150
W_gc_ (μM)	Grating area width	2
α°	Inclination1	55
β°	Inclination2	110
Λ (μM)	Grating period	1.04
Dc	Grating duty cycle	0.4
SiN thickness (μM)	SiN layer thickness	0.35
L_Taper_ (μM)	Taper length	11

**Table 2:** Performance comparison table for optical antennas.

	Element Size	Working Bandwidth	Emission Efficiency (At Center Working Wavelength)
This work	2 μM × 150 μM	500 nm (1 dB)	73%
CN (2021) [18]	7.6 μM × 4.5 μM	230 nm (1 dB)	89%
NRCS (2020) [17]	3.65 mm	N.A.	72%
UCAS (2019) [15]	1.5 μM × 80 μM	140 nm (3 dB)	94%
MIT (2017) [16]	3 mm	N.A.	93%
MIT (2013) [13]	9 μM × 9 μM	N.A.	51%

## Data Availability

All raw data generated in the production of this manuscript are available on request.

## References

[1] Dostart N., Zhang B., Khilo A., Brand M., Qubaisi K.A., Onural D., Feldkhun D., Wagner K., Popović A. (2020). Serpentine optical phased arrays for scalable integrated photonic LIDAR beam steering. Optica.

[2] Fatemi R., Khachaturian A., Hajimiri A. (2019). A Nonuniform Sparse 2-D Large-FOV Optical Phased Array With a Low-Power PWM Drive. IEEE J. Solid-State Circuits.

[3] Miller S.A., Chang Y.-C., Phare C.T., Shin M.C., Zadka M., Roberts S.P., Stern B., Ji X., Mohanty A., Gordillo O.A.J. (2020). Large-scale optical phased array using a low-power multi-pass silicon photonic platform. Optica.

[4] Huang M.C.Y., Zhou Y., Chang-Hasnain C.J. (2007). A surface-emitting laser incorporating a high-index-contrast subwavelength grating. Nat. Photonics.

[5] Roelkens G., Vermeulen D., Van Laere F., Selvaraja S., Scheerlinck S., Taillaert D., Bogaerts W., Dumon P., Van Thourhout D., Baets R. (2010). Bridging the gap between nanophotonic waveguide circuits and single mode optical fibers using diffractive grating structures. J. Nano Nanotechnol..

[6] Zhu L., Kapraun J., Ferrara J., Chang-Hasnain C. (2015). Flexible photonic metastructures for tunable coloration. Optica.

[7] Zhu L., Yang W., Chang-Hasnain C. (2017). Very high efficiency optical coupler for silicon nanophotonic waveguide and single mode optical fiber. Opt. Express.

[8] Karagodsky V., Sedgwick F.G., Chang-Hasnain C.J. (2010). Theoretical analysis of subwavelength high contrast grating reflectors. Opt. Express.

[9] Poulton C.V., Byrd M.J., Raval M., Su Z., Li N., Timurdogan E., Coolbaugh D., Vermeulen D., Watts M.R. (2017). Large-scale silicon nitride nanophotonic phased arrays at infrared and visible wavelengths. Opt. Lett..

[10] Chung S., Abediasl H., Hashemi H. (2018). A Monolithically Integrated Large-Scale Optical Phased Array in Silicon-on-Insulator CMOS. IEEE J. Solid-State Circuits.

[11] Doylend J.K., Heck M.J., Bovington J.T., Peters J.D., Coldren L.A., Bowers J.E. (2011). Two-dimensional free-space beam steering with an optical phased array on silicon-on-insulator. Opt. Express.

[12] Van Acoleyen K., Komorowska K., Bogaerts W., Baets R. (2011). One-Dimensional Off-Chip Beam Steering and Shaping Using Optical Phased Arrays on Silicon-on-Insulator. J. Lightwave Technol..

[13] Sun J., Timurdogan E., Yaacobi A., Hosseini E.S., Watts M.R. (2013). Large-scale nanophotonic phased array. Nature.

[14] Chang-Hasnain C.J., Yang W. (2012). High-contrast gratings for integrated optoelectronics. Adv. Opt. Photonics.

[15] Wang P.F., Luo G.Z., Yu H.Y., Li Y.J., Wang M.Q., Zhou X.L., Chen W.X., Zhang Y.J., Pan J.Q. (2019). Improving the performance of optical antenna for optical phased arrays through high-contrast grating structure on SOI substrate. Opt. Express.

[16] Raval M., Poulton C.V., Watts M.R. (2017). Unidirectional waveguide grating antennas with uniform emission for optical phased arrays. Opt. Lett..

[17] Ginel-Moreno P., Pereira-Martín D., Hadij-ElHouati A., Ye W., Melati D., Xu D.-X., Janz S., Ortega-Moñux A., Wangüemert-Pérez G., Halir R. (2020). Highly efficient optical antenna with small beam divergence in silicon waveguides. Opt. Lett..

[18] Khajavi S., Melati D., Cheben P., Schmid J.H., Liu Q., Xu D.X., Ye W.N. (2021). Compact and highly-efficient broadband surface grating antenna on a silicon platform. Opt. Express.

[19] Chen X., Thomson D.J., Crudginton L., Khokhar A.Z., Reed G.T. (2017). Dual-etch apodised grating couplers for efficient fibre-chip coupling near 1310 nm wavelength. Opt. Express.

[20] Yin X., Jin J., Soljačić M., Peng C., Zhen B. (2020). Observation of topologically enabled unidirectional guided resonances. Nature.

[21] Cheng L., Mao S., Li Z., Han Y., Fu H.Y. (2020). Grating Couplers on Silicon Photonics: Design Principles, Emerging Trends and Practical Issues. Micromachines.

[22] Schrauwen J., Van Laere F., Van Thourhout D., Baets R. (2007). Focused-ion-beam fabrication of slanted grating couplers in silicon-on-insulator waveguides. IEEE Photonics Technol. Lett..

[23] Wang B., Jiang J., Nordin G.P. (2005). Embedded slanted grating for vertical coupling between fibers and silicon-on-insulator planar waveguides. IEEE Photonics Technol. Lett..

[24] Xie W., Komljenovic T., Huang J., Tran M., Davenport M., Torres A., Pintus P., Bowers J. (2019). Heterogeneous silicon photonics sensing for autonomous cars [Invited]. Opt. Express.

[25] Vogt M.R. (2016). Development of physical models for the simulation of optical properties of solar cell modules. Tech. Inf. (TIB).

[26] Chrostowski L., Hochberg M. (2015). Silicon Photonics Design: From Devices to Systems.

[27] Doerr C.R., Chen L., Chen Y., Buhl L.L. (2010). Wide Bandwidth Silicon Nitride Grating Coupler. IEEE Photonics Technol. Lett..

[28] Zhang Z., Kleinert M., Maese-Novo A., Irmscher G., Schwartz E., Zawadzki C., Keil N. (2014). Multicore polymer waveguides and multistep 45 mirrors for 3D photonic integration. IEEE Photonics Technol. Lett..

[29] Lee J.K., Lee S.H., Min J.H., Jang I.Y., Kim C.K., Moonet S.H. (2009). Oblique-directional plasma etching of Si using a Faraday cage. J. Electrochem. Soc..

[30] Stegmuller B., Westermeier H., Thulke W., Franz G., Sacher D. (1991). Surface emitting InGaAsP/InP distributed feedback laser diode at 1.53 mu m with monolithic integrated microlens. IEEE Photonics Technol. Lett..

[31] Boyd G.D., Coldren L.A., Storz F.G. (1980). Directional reactive ion etching at oblique angles. Appl. Phys. Lett..

[32] Schaepkens M., Oehrlein G.S. (2001). A review of SiO_2_ etching studies in inductively coupled fluorocarbon plasmas. J. Electrochem. Soc..

[33] Sheen D.M., Ali S.M., Abouzahra M.D., Kong J.A. (1990). Application of the three-dimensional finite-difference time-domain method to the analysis of planar microstrip circuits. IEEE Trans. Microw. Theory Tech..

[34] Oetting C.C., Klinkenbusch L. (2005). Near-to-Far-Field Transformation by a Time-Domain Spherical-Multipole Analysis. IEEE Trans. Antennas Propag..

